# Epigenetic Alterations under Oxidative Stress in Stem Cells

**DOI:** 10.1155/2022/6439097

**Published:** 2022-08-29

**Authors:** Min Huang, Qiang Wu, Zhi-Hong Jiang

**Affiliations:** State Key Laboratory of Quality Research in Chinese Medicine, Macau University of Science and Technology, Macau, SAR, China

## Abstract

Epigenetic regulation of gene expression, including DNA methylation and histone modifications, provides finely tuned responses for cells that undergo cellular environment changes. Abundant evidences have demonstrated the detrimental role of oxidative stress in various human pathogenesis since oxidative stress results from the imbalance between reactive oxygen species (ROS) accumulation and antioxidant defense system. Stem cells can self-renew themselves and meanwhile have the potential to differentiate into many other cell types. As some studies have described the effects of oxidative stress on homeostasis and cell fate decision of stem cells, epigenetic alterations have emerged crucial for mediating the stem cell behaviours under oxidative stress. Here, we review recent findings on the oxidative effects on DNA and histone modifications in stem cells. We propose that epigenetic alterations and oxidative stress may influence each other in stem cells.

## 1. Introduction

Oxidative stress is due to pathologically accumulated excessive free radicals in the body associated with increased lipid peroxidation. Reactive oxygen species (ROS) are broadly considered as a deleterious reactive by-product of oxidative phosphorylation (OXPHOS) through aerobic mitochondrial respiration, although appropriate concentration of harmless ROS is essential to intracellular signaling transduction [1]. Physiological ROS is heavily regulated by antioxidant enzymes, while verbose ROS production is caused by certain lesions. Consequently, the antioxidant defense system fails to scavenge the ROS in time since excessive oxygen free radicals are strong and unstable molecules that represent in varying valences, including singlet oxygen (^1^O_2_), super anion (O_2_^−^), the hydroxyl radical (OH^−^), and hydrogen peroxide (H_2_O_2_) [2]. These free radicals are reactive to display their inherent chemical properties, which confer reactivity to different biological processes. ROS are generally connected with oxidative stress, which comprehensively damages the cells from molecular to cellular level. Constant exposure to oxidative stress can not only irreversibly retrofit the functional structure of intracellular biomacromolecules but also ultimately result in permanent DNA lesions, leading to cellular damage, homeostasis impairment, and cell fate changes [3].

Chromatin represents the dynamic macromolecular complexes of DNA and histones that folds DNA sequences into eukaryotic nucleus. A nucleosome is a fundamental unit of chromatin that contains a 147 bp of DNA wrapped around the core histone octamers [4]. The N-terminal tails of histones are subjected to provide the reaction sites to undergo multiple posttranslational modifications (PTMs), including acetylation, methylation, phosphorylation and ubiquitylation, and SUMOylation [5]. These covalent modifications are mediated by a set of histone-modifying enzymes [6]. It is widely accepted that PTMs can alter the chromatin conformation into either opening state (euchromatin) or compaction state (heterochromatin) in order to activate/silence gene expression. Interestingly, histone PTMs can be altered by ROS-mediated oxidative stress [7] and plays a role in transcriptional regulation [8].

Human body is an endogenous system of regeneration mediated by tissue stem cells [9]. Stem cells are characterized by their unique properties: self-renewal and multipotency. The self-renewal allows stem cells to be sustained for prolonged periods while holding the potential to differentiate into other cell types [10]. Stem cells can also give rise to one or more cell type(s) which functionally reconstitute of the tissue of origin [11]. According to their origins, stem cells can be classified into two groups: embryonic stem cells (ESCs) and adult stem cells. ESCs are derived from the inner cell mass of blast-stage embryos. ES cells are pluripotent since they can differentiate into almost all cell types except placental cells [12–14]. In contrast to ESCs, adult stem cells are widely presented in many adult tissues. Adult stem cells are generally restricted to differentiate into a certain cell type as their original organs. It is appeared all stem cells are devoted to regulate a balanced ROS and oxidative stress that maintain their uncommitted state and avoid progressive genetic damages. Hence, understanding the redox biology of stem cells is significant to stem cell therapy in high quality.

This review is aimed at (1) underscoring the redox regulation of stem cell fate decision and (2) reviewing epigenetic alterations and oxidative stress in stem cells.

## 2. Redox Biology in the Cells

Continuous chemical fuel supplement in the form of ATP is necessary to functionally support essential biomacromolecule synthesis and cellular activities. With the catalysis of ATP synthase, electron transport chain (ETC) is remodeled from the reduced enzymes, such as NADH, and transported to molecular oxygen to from a high-throughput proton current [15]. ATP is mainly produced in mitochondria via TCA/oxidative phosphorylation but also can be generated by other metabolic pathways, such as glycolysis, fatty acid oxidation, and fermentation [16]. Activation of mitochondrial ETC produces powerful proton beam. However, meanwhile, it promotes the formation of ROS on the inner mitochondrial membranes during oxidative phosphorylation [17]. NADH family proteins are the major molecules which response to transfer electrons to the molecular oxygens, while the strong proton current results in creation of mitochondrial membrane potentials by recruiting large amount of positively charged photons from the mitochondrial matrix and transporting them to the intermembrane space [18]. With the assistance of enzymatic complexes, the protons are reciprocally transported to the mitochondrial matrix and alighted to produce a strong force that forms ATP.

In some circumstance, such as exposure of environmental oxygen tension, the electrons from ETC are abruptly blocked, and the partial reduction of molecular oxygen generates electrophilic superoxide anion radicals in the mitochondrial matrix [19]. Such successive generation of free radicals is thought to be mitochondrial ROS molecules. ROS can behave as a key signal molecules freely tramping between inter- and intracellular communication though emitting signal transduction [20]. Another recent view shows that ROS acts as an essential signal transductor that contributes to adapt the environmental cues in the evaluation process [21]. Indeed, ROS in prokaryotes activates the transcription factors for adaptation to stress [22].

Abnormal accumulation of cellular oxygen concentrations may result in an aggravate pressure known as oxidative stress. It is widely accepted that the oxidative stress is primarily caused by the imbalance between generation and elimination of ROS [23]. Under physiological conditions, a certain concentration of acceptable ROS level is maintained with a safe threshold. However, when cells are exposed in unfavorite circumstances, the level of ROS can be dramatically increased and break through the safe threshold [24]. To defense the excessive ROS, the antioxidant systems will rapidly be activated to reduce the ROS level back to the safe threshold. This process is considered as acute oxidative stress [25]. Of note, acute oxidative stress is a temporary pathological process that induces the oxidative damage to the cells within a short period. Yet, in some cases, such as antioxidant system failure or inactivation of antioxidant enzymes, the ROS level is not permanently neutralized. The substantial deposit of ROS no longer returns to the stationary threshold, and the high level of ROS becomes a chronic oxidative stress [26]. Unlike acute oxidative stress, chronic oxidative stress is shown as an irreversible hierarchical damage which can be maintained at a relative higher level. Indeed, chronical oxidative stress is defined as quasistationary level [27]. As the continuous load of oxidative stress, many pathological processes result from the associated oxidative stress which can be defined as a pathological term.

Cells must maintain a genetically stable genome against hostile endogenous and exogenous DNA damage agents. Toxic oxidate agents may endanger the genetic materials and result in abrupt upregulation of DNA lesions. In order to protect the genetic materials form DNA damage, cells employ DNA damage response (DDR) pathways that purge, delete, and reset the DNA lesions [28]. In eukaryotes, DNA damage and its repair mechanism are occurred in the context of chromatin. Upon recognizing the DNA damage, several key mediators of DDR are activated immediately to prevent the cumulation of cascading damages. These mediators work cooperatively to delete the errors in different aspects. The DNA lesions are preliminarily recognized by the sensor protein though inspecting lesion sites or chromatin alterations. The transducers are responded to initiate the repair action and transit the damage signal to the downstream effectors [29]. Failure to fix DNA errors may multifacetedly result in genetic mutation and deviant chromosomal remodelling [30]. Remarkably, a robust DDR is critical to maintain a redox homeostasis in stem cells since replication errors in stem cells not only inherit a detrimental genetic material to the progeny but also destroy their differentiation potentials [31]. For example, in mouse ESCs, p53 suppresses Nanog transcription though binding to its promoter. The inhibition of Nanog by p53 facilitates the elimination of damaged cells from the replicating stem cells, followed by the establishment of an efficient p53-dependent cell cycle arrest or apoptotic program in differentiated progeny cells [32].

Notably, many studies have demonstrated that ROS, mitochondria, and oxidative stress play important roles in apoptosis [33]. Conversely, antioxidants and thiol reductants can inhibit apoptosis [33]. For instance, increase of ROS level resulted from environmental toxicants can be harmful to oocyte mitochondria and may lead to mitochondria dysfunction and cellular apoptosis [34]. Bmi1 is important to regulate mitochondrial function and control ROS level in diverse cell types [35]. However, in T cells, depletion of Bcl-2 is not related to Bmi1 but can cause T cells be sensitized to apoptosis by ROS [36]. In addition, p62/SQSTM1 plays an important role in the antioxidant property of hepatocytes [37]. Interestingly, recent studies have shown that nanoparticles can be a useful mediator for ROS release specifically to cancer cells for cancer therapy. Stannic oxide nanoparticles (NPs) can induce oxidative stress, inhibit cell growth, and induce apoptosis of breast [38] and oral cancer cells [39]. However, a more in-depth study is needed to determine its roles. Bismuth oxide NPs can induce ROS generation and cytotoxicity, thus causing cancer cell apoptosis [40]. In contrary, oxidative stress and cytotoxicity induced by heavy metal Pb in human lung cells (A549) can be prevented by TiO_2_ nanoparticles [41]. Hence, it will be worth further research on different nanoparticles which can mediate oxidative stress in cancer therapy.

### 2.1. Oxidative Stress in Stem Cells

The dose of oxidative stress loaded in stem cells is critical to maintain their stem cell identity and homeostasis. Low concentration of ROS is essential for maintaining ESCs in undifferentiated state, while ROS-mediated oxidative stress can modulate stem cell differentiation, senescence, apoptosis, and repopulation [42]. In addition, appropriate level of ROS can accelerate reprogramming process [43]. Therefore, ROS signaling is critical to the stem cell fate decision.

It is noteworthy that pluripotent stem cells and adult stem cells have complete different metabolic programs. Pluripotent stem cells (PSCs) including embryonic stem cells (ESCs) and induced pluripotent stem cells (iPSCs) heavily reply on glycolysis as main energy source to meet their energy demands. Similarly, suppression of mitochondrial respiration is beneficial to maintain the pluripotency. Indeed, either promotion of glycolysis or suppression of oxidative phosphorylation can accelerate the reprogramming process during which somatic cells are converted into induced pluripotent stem cells (iPSCs) [44]. Considering the hypoxic condition in the uterus, it is not surprising that ESCs—the *in vitro* counterpart of inner cell mass in the early embryo—prefer glycolysis. ESCs indeed have less mitochondria mass with immature morphology and maintain their stemness at lower oxygen level [45]. Interestingly, human ESCs showed stronger resistance to oxidative and genotoxic stress as compared with somatic cells [46]. Consistently, pluripotent stem cells in naïve state present a relatively higher oxidative phosphorylation level in primed state [47]. In addition, most iPSC clones produced from aged tissue donors do not suppress the oxidative phosphorylation; only young iPSCs produced from young tissue donor and ESCs are able to reset the oxidative phosphorylation into glycolysis [48]. Hence, the dynamic equilibrium between the generation and elimination of ROS must be tightly regulated in pluripotent stem cells.

Although ESCs favor glycolysis to generate energy, regulating balanced ROS level is important to preserving mtDNA integrity in ESCs [49]. Compared with nuclear DNA, mtDNA has a relatively higher risk of mutation because they are structurally naked and have higher chance of replication errors [50]. The expression of mtDNA remains at a low level in ESCs, and it only shows a transient increase during ESC differentiation. In addition, the mitochondrial genome is spatially close to the inner mitochondrial membrane and thereby susceptible to the ROS damage [51]. The pathogenic mtDNA molecules result in aberrant mitochondrial respiration. Therefore, an optimal ROS is important to functionally preserve the normal mtDNA integrity in ESCs.

In order to maintain the genomic stability, the stress control and DNA repair in ESCs are particularly more stringent, compared with differentiated cells [52], while DNA repair capability may gradually loss during development. The high proliferation of ESC is attributed to the distinct cell cycle where the G1 phase is much shorter than differentiated cells since ESCs do not suffer from replicative senescence [53]. Like many cells, ESCs are exquisitely sensitive to the oxidative toxicity which can directly alter their regular cell cycle. Under high oxygen tension, ESCs shortly enter the cell cycle arrest state at G2/M phase although they can establish an expeditious recovery machinery to restore the normal cell cycle [54]. In addition, increased mitochondrial ROS production retards the cell proliferation rate with longer S phase [55]. Taken together, these studies suggest that endogenous ROS regulates ES cell propagation through modulating their cell cycle progression.

Though ESCs are originally resistant to the oxidative stress due to their recovery machinery of cell cycle, high level of oxidative phosphorylation in ESCs not only gradually results in ESC differentiation but also induces apoptosis [56]. Several studies have demonstrated the benign effects of elevated ROS during cardiac differentiation. At pluripotent state, ROS degrades the SIRT1 deacetylase by recruiting the FOXO proteins [57], which is a key family that regulates the master pluripotent genes in human ESCs [56]. During differentiation, acute eruption of ROS promotes ESC development towards cardiomyocyte phenotype, while chronic treatment of H_2_O_2_ terminates the differentiation process [58]. Oxidative stress further activates the Notch1 pathway and hence enhances the cardiogenic lineage specification [59].

Adult stem cells, such as mesenchymal stem cells (MSCs), hematopoietic stem cells (HSCs), and neural stem cells (NSCs), are more sensitive to physiological oxygen tension than pluripotent stem cells. Indeed, MSCs cultured in an atmospheric oxygen show a low cell proliferation with accelerating senescence in comparison with the physiological oxygen [60]. Similarly, exposure of HSCs to an environmental oxygen tension may induce extra physiologic oxygen shock/stress [61]. Prolonged quiescent HSCs barely need cellular ROS, while active HSCs require endogenous ROS for proper differentiation [62]. Approximately 1% of low oxygen condition is advantageous to maintain the human c-Kit+ cardiac progenitor cell survival and expansion [63]. In addition, the concentration of intracellular ROS varies in different state of the adult stem cells. For instance, embryonic neural stem cells have slightly higher cellular ROS level to support their stem cell survival and expansion and further increase when stem cells transit from proliferation to differentiation [64]. The ROS level in MSCs, another examples, is tightly regulated in an extremely low stage in undifferentiated state while relatively enhanced during their differentiation into adipocytes [65]. Analogous study also suggests that high level of ROS with addition of exogenous H_2_O_2_ promotes the redirectional differentiation of MSCs into adipocytes [66]. In fact, the suppression of mitochondrial metabolism decelerates the adipogenic differentiation process because reduction of mitochondrial respiration diminishes the production of ROS [67]. Furthermore, increased ROS level is essential to support the chondrogenesis in ATDC5 cells and primary chondrocytes derived from mouse embryos [68]. These evidences concordantly demonstrate that ROS and oxidative stress are necessary for stemness and differentiation of adult stem cells.

### 2.2. Oxidative Stress and DNA

DNA methylation and histone modifications are the most well-understood epigenetic mechanisms. DNA methylation occurs on CpG dinucleotide and mainly mediates gene silencing. The addition of methyl groups by DNA methyltransferases (DNMTs) alters the structure of the major groove of DNA where the proteins attach, leading to heritable changes in the chromatin structure [69, 70]. In early embryonic development, lineage-specific genomic methylation patterns are created by *de novo* transferases DNMT3A and DNMT3B. The established patterns are further copied by cell divisions by the preservation of DNMT1. Loss of DNMT3A in iPSCs affects the physiological cardiomyocyte homeostasis though activating PPAR*γ*, where it accumulates with lipid vacuoles [71]. Mice with DNMT3B aberrant methylation are featured as neural disorder [72].

DNA constituents are especially susceptible to the damaging effects of ROS, such as 8-hydroxyguanine (8-oxo-dG), which is the most familiarly generated base lesion and a good measure of oxidative damage in DNA. Upon oxidative stress, the hydroxyl radical reacts with the guanine (G) sites of DNA sequence which is the most sensitive to oxidative stress out of the four bases in DNA. 8-oxo-dG further destroys other bases to cause oxidation where occurs G ➔ T mutations [73]. Such a transversion caused by ROS is believed as inherently mutant site on a nascent strand, and the presence of 8-oxo-dG may give an undesirable methylation to the adjacent sites, such as cytosine bases from the damaged guanine [74, 75]. A relatively high dose of 8-oxo-dG in the DNA seemly tends to change various stem cell activities. In mouse ESCs, a steady-level of 8-oxo-dG theoretically can arise a considerable amount of mutation sties after cell division [76]. A recent study reports that oxidative stress generated from immune cells can suppress the DNMT activity in proliferating cells [77]. Consistently, a genome-wide study showed that oxidative damage can induce an inheritable mutated DNA methylation pattern and sublethal dose of hydrogen peroxide is enough to damage DNA methylation patterns in proliferation cells [78]. Deletion of DGCR8 in MSCs is associated with a hypermethylation in the CpG island upstream of SOD2, leading to slow proliferation rate with increased ROS level [79].

During embryonic development, a key epigenetic factor known as ten-eleven translocation (TET) can reprogram DNA methylation pattern. In mouse ESCs, Tet1 is acquired for ES cell maintenance, and downregulation of Tet1 correlatively suppresses DNA methylation of *Nanog* promoter, indicating a causative role of Tet1 in DNA methylation [80]. Interestingly, the TET proteins can successively oxidize 5-methylcytosine (5-mc) to 5-hydroxymethylcytosine (5-hmC) [81] as a resultant of demethylation of CpG site modification [82] and subsequently into 5-formylcytosine and 5-carboxylcytosine [83]. All 5-mc oxidation products behave as the intermediates which response to the conversion of 5-mc to the unmodified cytosines. In advanced pronuclear-stage zygotes of mouse embryogenesis, the paternal pronucleus has a certain level of 5-hmC but low level of 5-mC. Importantly, deficiency of Tet3 may erase both 5-hmC and 5-mC pattern [84].

Like methylation of nuclear DNA, mtDNA can also methylated into 5-mC in presence of mtDNMT1. Cells with the ample energy demand are expected to increase mitochondria mass with a relatively high copied number of mtDNA, which is used to monitor the mtDNA methylation state. Deletion of mtDNA may change nuclear genome which is caused by damaging the potential of oxidative damage recovery [85]. In addition, a proportion of global DNA methylation is potentially mtDNA methylation [86]. In mammals, redox stress contributes to activate a set of nuclear-encoded transcription factors, such as PCG1alpha and NRF1 [87]. The PCG1alpha/NRF1 complex positively regulates the TFAM transcription and ordinates the core ETC complexes, which imply that mitochondria proper may also regulate the cytosine methylation level [88]. Therefore, it appears that there is a delicate interrelationship between nuclear and mitochondria. For instance, 5-hmc can be detected in mtDNA and TET proteins can be also identified in the mitochondrial protein fractions [89]. TET proteins can interact with several intermediates of Krebs cycle, such as JmjC and alpha-ketoglutarate, that serve as a potent substrate of mitochondrial ROS production. Therefore, mitochondrial integration can influence DNA methylation by multifaced aspects, either by modulating the ROS level or regulating the intermediates of Krebs cycle, and thereby regulate the DNMTs and TET proteins [90].

mtDNA methylation is a critical epigenetic regulation in mitochondrial activity in stem cells. The mitochondrial methylation level is downregulated in aged human cardiac mesenchymal stem cells [91]. By profiling the CpG methylation status across the mitochondrial genome, researchers showed that aged MSCs treated with chronic oxidated stress appear to show a global mtDNA hypomethylation [92]. In turn, the nuclear *de novo* methyltransferase level can disturb the mitochondrial biogenesis. In human ESCs, loss of DNMT3B destroys the mitochondrial fission and fusion balance, leading to decreased mtDNA level and transition from the glycolytic metabolism to oxidative phosphorylation [93]. Indeed, ESCs that have different DNA haplotypes display multiple gene activities associated with stemness, DNA methyltransferases, and energy metabolism [94]. Overall, these results show that mitochondrial DNA methylation significantly contributes to global methylation pattern in stem cells.

In all, mitochondrial DNA methylation has been recognized as an emerging DNA methylation pattern that is sensitive to oxidative stress. Mitochondrial ROS production modulates the epigenetic prolife in both nuclear DNA and mitochondrial DNA. Thus, it is of great interest to further study the correlations among mitochondrial DNA methylation, genomic DNA methylation, and oxidative stress.

### 2.3. Oxidative Stress and Histone (De)acetylation in Stem Cells

Histone acetylation is mediated by histone acetyltransferases (HATs). It is generally associated with open chromatin and gene activation. On the contrary, histone deacetylation is mediated by histone deacetylases (HDACs) that condenses the chromatin and inhibits transcriptional activities [95]. Hyperacetylation on the N-terminal tail of histones H3 and histone H4 has a strong propensity to activate the gene expression through unfastening the chromatin condensation and thereby increasing the accessibility of DNA to transcription factors. There are three HAT families: p300/CBP, GNAT, and MYST [96]. To date, a total of 18 mammalian HDACs have been identified and categorized into four classes in accordance with their homology to yeast HDACs. Class I HDACs are mainly to regulate the cell growth and death, and class II HDACs are predominantly in lineage specification during embryonic development. NAD+-dependent class III HDACs are responsible for sirtuin-associated metabolism. Class IV HDAC (only one member HDAC11) is restricted in several organs, such as kidneys, testis, and brain [97].

The redox state of cells contributes to regulate chromatin remodelling and mediate gene expression. It is widely accepted that increased oxidative stress is implicated to enhance histone acetylation, particularly in acetylation of histone H3 and H4. High oxidative stress level has been reported to alter the hyperacetylation of histone proteins by activation of nuclear factor kappa B in human alveolar epithelial cells [98]. ROS production has been shown to control the histone acetylation differentially in various cell types [99]. A recent study shows that an increased mitochondrial ROS caused by Sod2 downregulation in stromal precursor cells inhibits differentiation potentials of osteogenesis and adipogenesis and induces chromatin condensation with a reduction of histone H3K27 acetylation [100]. Indeed, high level of SOD2 causes increased ROS level and facilitates histone H3 acetylation though promoting the recruitment of p300/CBP [101]. On the other hand, p300/CBP can also acetylate histone H3K9 to increase the ROS production [90]. Therefore, HAT p300/CBP complex appears to dynamically regulate the balance of ROS in cells.

Acetylation state in mitochondrial proteins is determinant to the mitochondrial function. Differentiation of MSCs into osteoblast differentiation is accompanied with increasing KLF2 level which activates histone H3K27ac and H3K4me3, during which mitochondrial ROS is suppressed [102]. The acetylation level modulated by oxidative stress was recently shown to determine the HSC quiescence by regulating SRC expression [103]. SRC3 is not only essential to maintain the quiescence status of HSCs by the prevention of ROS accumulation but also retains the acetylation level though direct recruitment of GCN5 to its promoter region. In mice model, high level of GCN5 can prevent the overactivated mitochondrial metabolism which may give excessive production of ROS induced by SRC3 deficiency, thereby rescuing the cell from oxidative damage [104]. During mammalian development, Hat1 is responsible for catalysing histone H4 on lys 5 and 12 in chromatin remodelling. Partially knocking down MEFs is shown to induce redox damage with increased the sensitivity to ROS level, leading to mitochondria developmental failure [105].

ROS can also change the regular molecular mechanisms of HDACs, particularly in class I and II HDACs. The condensed chromatin structure mediated by HDACs can be relaxed by ROS-mediated alterations, thus activating the aberrant gene expression [106]. More seriously, the oxidative damage mediated by other redox stress, such as lipid peroxides, may further result in carbonylation of class I HDACs [107]. Under pathophysiological oxidative stress, covalently modified cysteine residues of the HDAC1 can cause the decrease of histone deacetylase activity. In addition, the activity of HDAC2 can be downregulated by undergoing hypophosphorylation in high oxygen tension [108]. In mouse model, Sirt3 belonged to class III HDACs and can reduce ROS level though deacetylating Foxo3 [109]. Sirt3 can also stimulate antioxidant enzymes by activating the NF-*κ*B pathway [110]. Collectively, these evidences suggest that oxidative stress related to HDACs may attenuate their activity and expression and initiate the gene expression.

Proper regulation of HDACs decisively contributes to maintain a steady homeostasis in stem cells. SIRT1, the most conserved NAD+-dependent protein deacetylase, is involved in various stem cell activities. Sirt1 regulates mouse ESC differentiation through regulating the deacetylation of CRABPII [111]. In human MSCs, SIRT1 is supposed to regulate RUNX2 transcription which was induced by deacetylation of FOXO3 [112]. Suppression of SIRT1 leads to impairment of adipogenic/osteoblastic lineage commitment. Interestingly, the oxygen tension may change the stem cell characteristics, and subtle change in redox stress change found in different brain pathologies controls the cell fate of NPCs through histone deacetylase (HDAC) SIRT1 [113]. SIRT3 is also correlated to antioxidant enzyme activities. Upregulation of SIRT3 supports the human MSC to against the high oxidative stress. Conversely, loss of SIRT3 in human MSCs stimulates the cellular susceptibility to oxidative stress [114].

Overall, oxidative stress largely affects HDAC-mediated proliferation and differentiation in stem cells.

### 2.4. Oxidative Stress and Histone (De)methylation in Stem Cells

Unlike histone acetylation, methylation of histone proteins can be associated with either activation or silence of gene transcription. Histone methylation is catalysed by histone methyltransferases (HMTs) on specific histone lysine/arginine residues, particularly in histone H3 and H4, contributing to the high order of the chromatin structures. Histone H3K4me3 marks active promoters, while histone H3K9me3 and H3K27me3 silenced genes. Histone H3K27me3 is important in stem cells as it marks many silenced lineage genes [115–118]. Gene silence mark H3K9me3 is thought to positively correlate to the degree of adjacent lysine residues on H3 [119]. The reversible removal of histone methylation marks is mediated by histone demethylases [120]. Interestingly, a histone demethylase, JHDM1, can oxidatively catalyse H3K36me2 by configurating its evolutionarily conserved JmjC domain, which can induce radical-based oxidative reactions and catalyse methylation of trimethylated substrates [121]. Therefore, oxidative stress level can change histone methylation status through modulating the level of HMTs. Notably, both JmjC and LSD1 require the cofactors to mediate de methylation. LSD1-mediated demethylation via a FAD-dependent oxidative reaction suggests that methylation of histone lysine can be removed by oxidative histone demethylases [120]. JmjC domain proteins require iron (II), molecular oxygen, and ą-ketoglutarate and are relatively highly sensitive to oxidative stress [122]. The free radicals oxidize the iron (II) to (III) in the catalytical centre of histone demethylases and suppress their activity, hence inducing histone hypermethylation.

Several studies have demonstrated that aberrant methylation on histone can regulate the lineage differentiation of bone marrow-derived MSCs [123]. EZH2 also known as KMT6A catalyses the addition of methyl groups to H3K27, and it selectively promotes the adipogenesis with suppression of osteogenic lineages during MSC differentiation [124]. EZH2 is recently reported that it behaves as a suppressor of the antioxidant defense system in MSCs. EZH2 can enhance the level of H3K27me3 on the promoter of *Foxo1* and suppress its function to activate the downstream genes in antioxidant defense, leading to oxidative damage [125].

### 2.5. Epigenetic Control of Oxidative Stress in Embryogenesis

Genes are inherited from parental genomes and subsequently remodelled their specific sequence during meiosis. The recombined sequence remains unaltered through cell divisions, only when there is a replication error or harmful external exogenous source. Any epigenetic alteration presented in the genetic materials is correlated with the states of the gene activity during embryogenesis. The ovary is thought to a metabolically activated organ where the embryo faces oxygen paradox. A carefully balanced redox condition is a necessary prerequisite for early embryo development. Oxidative level in maternal reproductive system is associated with tissue remodelling, steady hormone maintenance, and cyclic endometrial regulation during menstruation. Undoubtedly, considerable production of ROS certainly facilitates the ovulation and germ cell identity [126]. During folliculogenesis, the involvement of acceptable oxidative tension promotes dominant follicle selection and ovulation induction [127]. However, involvement of superfluous ROS has been acknowledged as a decisive factor in early embryonic development. Evidence from human studies has shown that elevated oxidative DNA damage in sperm may reduce pregnancy rates [128]. High tension of seminal oxygen has also been correlated with sperm morphological defects [129] and rewrites the global methylation pattern in sperm, which in turn results in an increased preimplantation embryonic loss [130]. These evidences strongly suggest oxidative stress, and resulted epigenetic alterations play negative role in proper early embryogenesis. Nonetheless, given that epigenetic modifications are crucial to establish the correct epigenetic marks that regulate the physiological gene pattern at early stage of mammalian development, it is of great interest to further investigate histone modifications and DNA methylation/demethylation alterations in embryogenesis under oxidative stress.

## 3. Concluding Remarks

**Figure 1:**
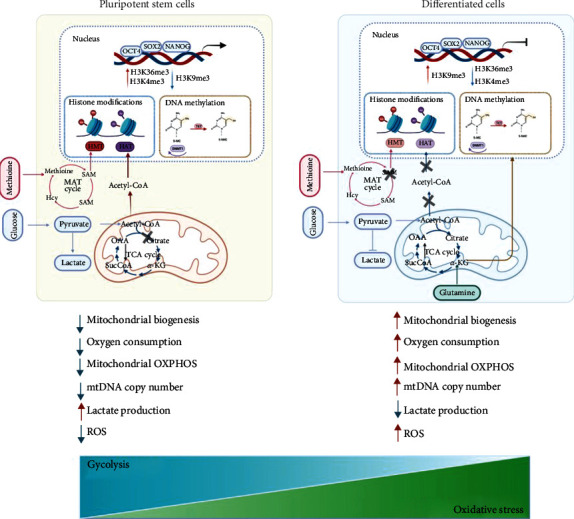
Epigenetic landscape modulated by oxidative stress in pluripotent stem cells and differentiated cells. OCT4, SOX2, and NANOG are core regulators to control cell identity in pluripotent stem cells. Histone marks H3K4me3 and H3K36me3 are located at pluripotent genes and H3K9me3 marks silenced genes. Histone proteins undergo a series of histone modifications, while DNA located at the CpG island is prone to occur DNA methylation. MAT cycle produces SAM which provides a methyl donor molecule utilized in histone methylation. Pluripotent stem cells (PSCs) heavily rely on glycolysis as the main the energy resource. In PSCs, most acetyl-CoA produced from TCA cycle are used for histone acetylation. The mitochondrial biogenesis is suppressed in PSCs. Subsequently, low level of *α*KG suppresses DNA methylation. In differentiated cells, OCT4, SOX2, and NANOG were suppressed. Loss of SAM inactivates histone methylation. Differentiated cells exhibit active aerobic respiration. Most acetyl-CoA enters the TCA cycle. Meanwhile, glutamine metabolism promotes high level of *α*KG that subsequently facilitates DNA methylation. HMT: histone methyltransferase; HAT: histone acetyltransferase; TET: ten-eleven translocation; DMT1: DNA methyltransferases 1; MAT: methionine adenosyltransferase; SAT: S-adenosyl methionine.

Recent studies have provided persuasive evidences that have highlighted the linkage of oxidative stress and epigenetic regulation in stem cells. Here, we illustrate the roles of oxidative stress in the evens of DNA methylation and histone PTMs (Figure 1). However, the epigenetic alterations under oxidative stress in stem cells have been poorly studied. More efforts are required to comprehensively understand the role of oxidative stress in stem cell epigenetics. For instance, epigenetics appears to delegate a complicated mechanism that involves various factors with ordination of multiple signal transduction. However, we lack ideal stem cell model system that simulates the nature of epigenetics in presence of ROS. High-throughput sequencing may help us to speculate the global changes in epigenetics caused by ROS. Apart from these issues, identifying novel epigenetic regulators can not only deepen our knowledge about oxidative stress in stem cells but also can provide new insights into stem cell therapy. Therefore, elucidating the molecular mechanism of epigenetic alterations mediated by oxidative stress in stem cells not only achieves the regenerative goals but also provides a novel and selective therapeutic approach to treat various diseases.

## Data Availability

Data used to support the findings of this study are included within the article.
